# Antibacterial and Antibiofilm Activity of Juglone Derivatives against *Enterococcus faecalis*: An *In Silico* and *In Vitro* Approach

**DOI:** 10.1155/2022/6197375

**Published:** 2022-11-10

**Authors:** Falak Niaz, Muhammad Faheem, Mutiullah Khattak, Iqra Anam Khawaja, Mi-Jeong Ahn, Umakanta Sarker, Syed Babar Jamal, Riaz Ullah, Atif Ali Khan Khalil

**Affiliations:** ^1^Department of Microbiology, Institute of Pathology and Diagnostic Medicine, Khyber Medical University, Peshawar, Pakistan; ^2^Department of Biological Sciences, National University of Medical Sciences, Rawalpindi, Pakistan; ^3^College of Pharmacy and Research Institute of Pharmaceutical Sciences, Gyeongsang National University, Jinju 52828, Republic of Korea; ^4^Department of Genetics and Plant Breeding, Faculty of Agriculture, Bangabandhu Sheikh Mujibur Rahman Agricultural University, Gazipur-1706, Bangladesh; ^5^Department of Pharmacognosy, College of Pharmacy, King Saud University, Riyadh 11421, Saudi Arabia; ^6^Department of Pharmacognosy, Institute of Pharmacy, Lahore College for Women University, Lahore, Pakistan

## Abstract

*Enterococcus faecalis* is a Gram-positive bacterium that is normally found in the gastrointestinal tract of humans and animals. *E. faecalis* is an opportunistic pathogen that causes a number of invasive and noninvasive infections. The emergence of multidrug resistance and biofilm formation by the bacterium have rendered the treatment of *E. faecalis* infections very difficult. Due its high rate of resistance and biofilm formation, there are very few options of treatment. Therefore, the current study was designed to evaluate the antibacterial and biofilm activities of juglone derivatives such as 2-methoxy-6-acetyl-7-methyljuglone and 2-ethoxy-6-acetyl-7-methyljuglone against multidrug-resistant (MDR) and biofilm-producing strains of *E. faecalis*. Agar well diffusion and broth microdilution methods were used to determine the antibacterial activities. Biofilm attachment and preformed biofilm inhibition were determined using crystal violet staining assay. Both juglone derivatives displayed promising antibacterial and antibiofilm activities against *E. faecalis*. Among these compounds, 2-ethoxy-6-acetyl-7-methyljuglone possessed better inhibitory activity with minimum inhibitory concentration (MIC) of 9.7 ± 3 *μ*M as compared to 2-methoxy-6-acetyl-7-methyljuglone (MIC, 19.5 ± 2 *μ*M). Additionally, 2-ethoxy-6-acetyl-7-methyljuglone also showed stronger antibiofilm activity than 2-methoxy-6-acetyl-7-methyljuglone. Furthermore, both the ligand molecules were docked into the binding site of the enterococcal surface protein, and the results revealed that both the molecules are actively binding in the target site. Based on these findings, juglone derivatives may be considered useful for the treatment of *E. faecalis* infections; however, further studies are required to elucidate the mechanism of action.

## 1. Introduction

*Enterococcus faecalis* is a noncapsulated, non-spore-forming Gram-positive bacterium, a member of group D Streptococci [1, 2]. Morphologically, these bacteria are round to oval in shape and arranged mostly in chains but are also found single or in pairs and groups [3, 4]. *E. faecalis* bacteria are found ubiquitously in the environment including dairy products, fermented food, plants, water, and soil and the intestinal tracts of animals [2]. Their ubiquitous presence is largely due to their ability to survive in various environmental conditions. They can grow in a wide temperature range of 10-45°C in the presence of high NaCl content and 40% bile and can survive for 30 min at 60°C [5]. Being a member of normal gastrointestinal, oral, and vaginal microbiota, *E. faecalis* can also become an opportunistic pathogen especially in hospitalized and immunocompromised individuals [6].

*E. faecalis* is responsible for 80−90% of hospital-acquired enterococcal infections [7]. A variety of virulence factors are responsible for the pathogenesis of *E. faecalis*, including cytolysin, aggregation substances, enterococcal surface protein (Esp), adhesins to collagen of *E. faecalis* (Ace), *E. faecalis* antigen A (EfaA), endocarditis and biofilm-associated pili (Ebp), and gelatinase and serine protease [5]. *E. faecalis* is an important *Enterococcus* that causes a range of infections in both humans and animals. Invasive infections include bacteremia endocarditis and peritonitis. Noninvasive infections include urinary tract infections and postsurgery wound infections [4].

*E. faecalis* possesses a high rate of antibiotic resistance with intrinsic resistance to cephalosporins and quinupristin-dalfopristin, and acquired resistance to quinolones, glycopeptides, and aminoglycosides by horizontal gene transfer (HGT) and mutation has led to the emergence of multi-drug-resistant (MDR) strains of this bacterium [8]. *E. faecalis* has the potential to inhabit the site of infection and secrete extracellular matrices that lead to biofilm development [9]. Biofilms have been linked to the development of chronic diseases that are difficult to treat with available antimicrobial drugs [10]. Biofilms exhibit strong resilience to antibiotics and other stressors, as well as a high rate of HGT and distinct gene expression patterns compared to planktonic bacteria [11]. Adaptive resistance to antibiotics, which is on the rise globally, poses a barrier to treating biofilm-associated chronic and acute infections [12]. Biofilm production increases resistance to antibiotics increased 100−1000-fold, and approximately 65−80% infections are mediated by biofilms [13, 14]. All these factors make *E. faecalis* treatment rather challenging; therefore, it is important to focus on newer therapeutic measures.

*Reynoutria japonica*, commonly called the Japanese knotweed, is a well-known medicinal plant that belongs to the family Polygonaceae [15]. The roots of *Reynoutria japonica* have been traditionally used for the treatment of infection, inflammation, jaundice, hyperlipidemia, and burns [16]. The roots of this plant are rich source of many biologically active compounds such as polydatin, emodin, resveratrol, physcion, and naphthoquinones [17]. Naphthoquinone and its derivatives especially juglone derivatives exert a variety of pharmacological activities [16, 17]. Recently, it has been reported that juglones such as 2-methoxy-6-acetyl-7-methyljuglone and 2-ethoxy-6-acetyl-7-methyljuglone show promising inhibitory activity against *Helicobacter pylori*^13^. Therefore, the current study was designed to evaluate the antibacterial and antibiofilm activities of these juglone derivatives against the MDR and biofilm-producing strains of *E. faecalis*. The bactericidal nature of these juglone derivatives was also determined.

## 2. Material and Methods

### 2.1. Two Juglone Derivatives

Two juglone derivatives, 2-methoxy-6-acetyl-7-methyljuglone and 2-ethoxy-6-acetyl-7-methyljuglone (MW, 260 and 284 g/mol, respectively), have been isolated from the ethanol extract of *Reynoutria japonica* Houtt. root in our laboratory. The isolation procedures and spectroscopic data have been already reported [13].

### 2.2. Isolates of Enterococci

A total of forty enterococcal isolates were collected from the microbiology laboratories of three tertiary care hospitals in Peshawar, Pakistan (Rehman Medical Institute, Northwest Hospital, and Khyber Teaching Hospital), from September 2020 to May 2021, after ethical approval was granted by the concerned ethical committees. The isolates were transferred in transport media in leak proof sterile container to the microbiology laboratory of the Institute of Basic Medical Sciences (IBMS), Khyber Medical University Peshawar, for further processing.

### 2.3. Identification of Enterococci

For validation and confirmation, the isolates were cultured on selective media (bile esculin agar) and identified by colony characteristics, Gram staining, growth on 6.5% NaCl media, catalase test, and esculin hydrolysis. The identification to species level was done using carbohydrate utilization tests (*E. faecalis* is positive for sorbitol and mannitol and *E. faecium* is positive for arabinose and raffinose utilization). Additionally, only *E. faecium* can grow at 4°C [18].

### 2.4. Antibiotic Susceptibility Test

To identify MDR strains of *E. faecalis*, antibiotic susceptibility test was performed. The modified Kirby-Bauer disc diffusion method was used according to the recommended Clinical and Laboratory Standard Institute (CLSI) guidelines. Antimicrobial susceptibility pattern of *E. faecalis* isolates was evaluated against the following antibiotic discs of amoxicillin (25 *μ*g), ciprofloxacin (5 *μ*g), erythromycin (15 *μ*g), gentamicin (15 *μ*g), tetracycline (30 *μ*g), vancomycin (30 *μ*g), fosfomycin (200 *μ*g), and linezolid (30 *μ*g). For control, *E. faecalis* ATCC 29212 strain was used. The results were interpreted according to the CLSI guidelines 2020.

### 2.5. Biofilm Assay

A fresh overnight *E. faecalis* culture was suspended in 5 mL of Luria-Bertani (LB) broth and incubated aerobically at 37°C for 24 h ^19^. After incubation tubes were centrifuged for 3-5 min, supernatant was discarded. The precipitate was suspended in normal saline and adjusted to 0.5 McFarland standard. To each well with 150 *μ*L of LB broth of 96-well polystyrene microtiter plate, 50 *μ*L of 0.5 McFarland cell suspension was added. *E. faecalis* TX-82 was used as a positive control while LB broth without inoculum was used as negative control. After 48 h of aerobic incubation, the plate was washed three times with phosphate-buffered saline and stained with 1% crystal violet for 15 min at room temperature (25°C). The quantification of biofilm was measured at OD 570 nm after solubilization of biofilm with 95% ethanol [19]. The result of the biofilm was interpreted as follows:

- ODsample ≤ ODcontrol: no biofilm
- ODcontrol < ODsample ≤ 2 × ODcontrol: weak biofilm
- 2 × ODcontrol < ODsample ≤ 4 × ODcontrol: moderate biofilm
- 4 × ODcontrol < ODsample: strong biofilm

### 2.6. Agar Well Diffusion Method

To test the efficacy of juglone derivatives as antibacterial agents against MDR and biofilm-producing strains of *E. faecalis*, the agar well diffusion technique was utilized. ATCC 29212 antibiotic sensitive strain was used as a control. The Mueller-Hinton agar plate was swabbed with 0.5 McFarland bacterial suspension with the help of sterile cotton swab. Wells (8 mm diameter and 2.5 cm apart) were made in the lawn with the help of sterile cork borer. Stock solutions of tested compounds were prepared in DMSO at a concentration of 10 mM and diluted further to the lower concentrations. One hundred microliter of sample solutions was added to the test wells, and DMSO was added as a negative control. After incubation for 24 h at 37°C in an aerobic environment, the inhibition zone was measured [19, 20].

### 2.7. Minimum Inhibitory Concentration (MIC)

The antibacterial activity of tested compounds was determined with modified microbroth dilution method. The MIC assay was determined using the method described earlier with some modifications [21]. A standard bacterial inoculum of 0.5 McFarland was prepared in tryptic soya broth. Two hundred microliter of broth was added to each well of a 96-well plate, and 100 *μ*L of 0.5 McFarland bacterial suspension was added to each well. Juglone derivatives were serial-double fold diluted and added to the well in different concentrations followed by incubation at 37°C for 24 h. After incubation, the MIC was defined as the lowest concentration of juglone derivatives which inhibit the growth of *E. faecalis.* All the assays were performed in triplicate.

### 2.8. Minimum Bactericidal Concentration (MBC)

To determine the MBC of juglone derivatives, 2 *μ*L of each suspension from the 96-well plate streaked on the blood agar plate along with the positive and negative controls. The plate was incubated aerobically at 37°C for 24 h, and the results for growth were observed [9]. MBC is the lowest concentration of molecules at which no growth is observed after incubation. No visible growth signified the bactericidal nature of juglone derivatives.

### 2.9. Biofilm Attachment Inhibition Assay

The standard 0.5 McFarland bacterial suspension was prepared by adding fresh *E. faecalis* colonies into 5 mL of LB broth. To the 96-well plate, 100 *μ*L of bacterial suspension was added. Sterile distilled water and bacterial inoculum were added for positive and negative controls, respectively. To the test the wells, 100 *μ*L of juglone derivatives were added in different concentrations, i.e., from higher to lower. The plate was incubated for 24 h at 37°C without shaking. The plate was washed three times with phosphate buffer and quantified using crystal violet staining assay as described earlier [22].

### 2.10. Preformed Biofilm Inhibition Assay

Standard bacterial suspension of 100 *μ*L was added to the 96-well plate along with the positive and negative controls and incubated aerobically at 37°C for 24 h. After incubation, juglone derivatives were added in different concentrations in amount of 100 *μ*L to test wells and incubated. In the next day, the plate was washed three times with phosphate buffer and stained with 1% crystal violet as describer earlier. All the assays were performed in triplicate for the purpose of validity and authenticity. For the result interpretation, the following formula was used to determine the inhibitory ability of molecules and expressed in percentage. The absorbance was measured spectrophotometrically at 570 nm [23].

(1) $$\begin{array}{c} \text{Percentage inhibition}=100-\left[\left\{\text{OD of test wells with}\frac{\text{sample}}{\text{OD}}\text{of negative control without sample}\right\}\times 100\right]. \end{array}$$

### 2.11. Statistical Analysis

All data of the present study were obtained from at least triplicate assays performed on different days. The data are given with the mean value followed by the standard deviation.

### 2.12. Molecular Docking

For the purpose of carrying out molecular docking, the MOE (Molecular Operating Environment) software package was utilized (http://www.chemcomp.com/). The Chemical Computing Group developed MOE with the intention of supporting cheminformatics, molecular modelling, bioinformatics, virtual screening, and structure-based drug design. Additionally, MOE can be used to build new applications that are based on SVL (Scientific Vector Language). The ligPlot implementation in MOE was utilized in order to make a prediction regarding the interaction of the enterococcal surface protein (Esp) with ligand molecules [24].

### 2.13. Ligand Preparation

The ligands for enterococcal surface protein (Esp) were constructed using MOE builder application [25]. Using the energy minimization algorithm found in the MOE tool, we were able to reduce the amount of energy that all of the ligand molecules had. For the purpose of minimizing the amount of energy required, the following parameters were utilized: gradient: 0.05, force field: MMFF94X, and chiral constraint: current geometry. The .mdb file format was used to save all of the minimized versions of the molecules. The next step involved using the prepared ligands as input files for the MOE docking program [24, 26].

### 2.14. Protein Preparation

Our research relied on the protein molecules enterococcal surface protein (Esp; PDB ID: 6ori), which could be found in the database known as Protein Data Bank. Following the elimination of water molecules, the three-dimensional protonation of the protein molecule was carried out. The energy minimization algorithm of the MOE tool was utilized to bring the energies of the protein molecules down to their lowest possible levels. For the purpose of minimizing the amount of energy required, the following parameters were utilized: gradient: 0.05, force field: MMFF94X+solvation, and chiral constraint: current geometry. When the root mean square gradient dropped below the 0.05 threshold, the energy minimization process came to an end. Docking was modelled after the minimized structures, which served as the template [26].

### 2.15. Docking

The binding of the ligand molecules with the protein molecules was analyzed using MOE docking program to find the correct conformation of the ligands, so as to obtain minimum energy structure. After the completion of docking, we analyze the best poses for hydrogen bonding/*π* − *π* interactions and root mean square deviation (RMSD) calculation by using MOE applications [24].

## 3. Results

### 3.1. Collection and Confirmation of *E. faecalis* Strains

Among the samples, 70% (*n* = 28) were *E. faecalis* and 30% (*n* = 12) were *E. faecium.*

### 3.2. Antimicrobial Susceptibility Testing

**Figure 1:**
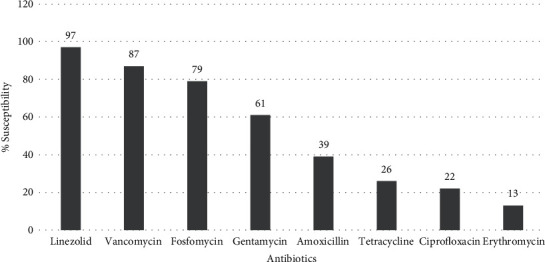
Antimicrobial susceptibility pattern of *E. faecalis.*

The modified Kirby-Bauer disc diffusion method was performed to identify the MDR strains of *E. faecalis*. Strains of *E. faecalis* that exhibited resistance to three or more antibiotics were categorized as MDR. Among the tested antibiotics, erythromycin showed the lowest potency, followed by ciprofloxacin and tetracycline with 13%, 22%, and 26% sensitivity, respectively. The most potent antibiotics against *E. faecalis* were linezolid, vancomycin, and fosfomycin, with 97%, 87%, and 79% sensitivity, respectively. Gentamycin and amoxicillin showed moderate activity, with 61% and 39% sensitivity, respectively (Figure 1).

### 3.3. Antibacterial Activity Using Agar of Juglone Derivative Well Diffusion Method

The antibacterial activities of juglone derivatives against the MDR biofilm-producing strains of *E. faecalis* and ATCC 29212 were evaluated using the agar well diffusion method. It was observed that 10 mM of 2-methoxy-6-acetyl-7-methyljuglone produced inhibitory zones of 15 ± 1 mm and 16.2 ± 0.8 mm against the MDR biofilm-producing strains of *E. faecalis* and ATCC 29212 strain, respectively; a decrease in the zone of inhibition was observed with a decrease in concentration. Furthermore, 2-ethoxy-6-acetyl-7-methyljuglone showed better inhibitory activity than the other juglone with inhibitory zones of 17.3 ± 0.5 mm and 18.1 ± 0.9 mm MDR strains and ATCC strain, respectively, at 10 mM concentration (Table 1).

### 3.4. Minimum Inhibitory Concentration of Juglone Derivatives

Microbroth dilution technique was used for the determination of minimum inhibitory concentration (MIC) of juglone derivatives. These compounds were checked against three MDR biofilm-forming strains (for validation) and the ATCC strain. The MICs of 2-ethoxy-6-acetyl-7-methyljuglone and of 2-methoxy-6-acetyl-7-methyljuglone were 9.7 ± 3 and 19.5 ± 2 *μ*M, respectively (Table 2). According to these results, 2-ethoxy-6-acetyl-7-methyljuglone was more potent against MDR *E. faecalis* and ATCC strains than 2-methoxy-6-acetyl-7-methyljuglone. Based on the minimum bactericidal concentration (MBC), testing both juglone derivatives was found to be bactericidal.

### 3.5. Biofilm Attachment Inhibition Assay

**Figure 2:**
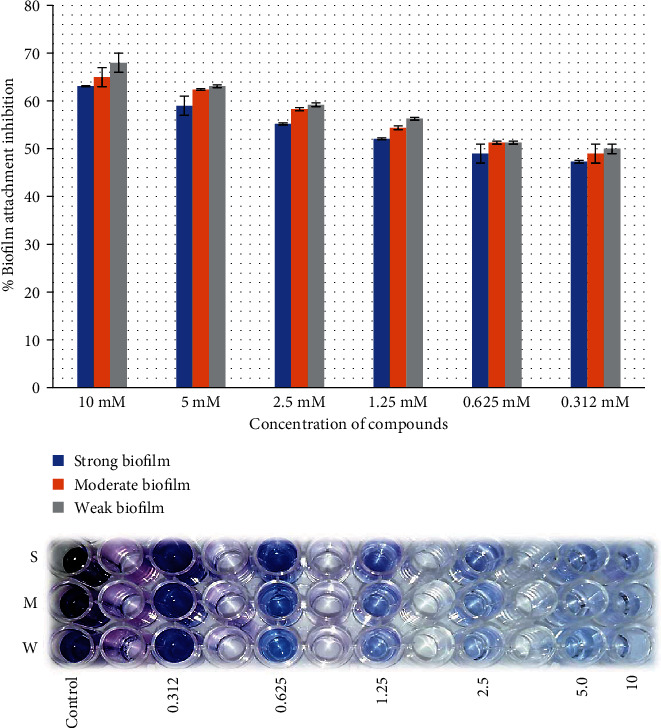
Effect of 2-methoxy-6-acetyl-7-methyljuglone on the biofilm formation of MDR *E. faecalis* as assessed by crystal violet staining. The bar graph indicates the percentage of attachment biofilm inhibition. S: strong; M: medium; W: weak biofilm producer.

Both juglones showed promising initial cell attachment inhibition activity. In particular, 2-ethoxy-6-acetyl-7-methyljuglone showed better biofilm attachment inhibition rate of 70% against strong biofilm producer as compared to 2-methoxy-6-acetyl-7-methyljuglone which showed inhibition rate of 63.1%. The same results were observed against moderate (74.1%) and low (76.1%) biofilm producers, with 2-ethoxy-6-acetyl-7-methyljuglone showing a higher rate of inhibition than 2-methoxy-6-acetyl-7-methyljuglone (68% and 65%, respectively).  Figure 2 shows the dose-dependent biofilm inhibition of *E. faecalis.*

### 3.6. Preformed Biofilm Inhibition Assay

**Figure 3:**
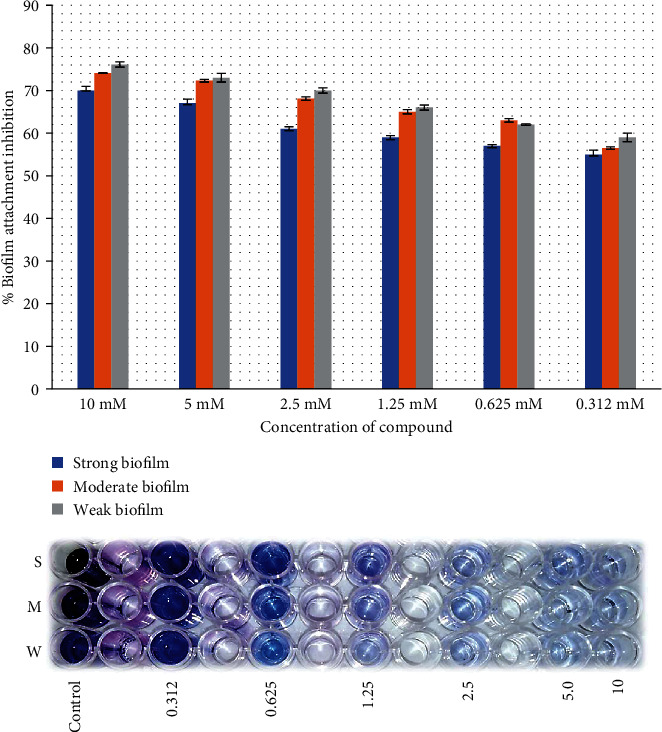
Effect of 2-ethoxy-6-acetyl-7-methyljuglone on the preformed biofilm of MDR *E. faecalis* as assessed by crystal violet staining. The bar graph indicates the percentage of preformed biofilm inhibition. S: strong; M: medium; W: weak biofilm producer.

**Figure 4:**
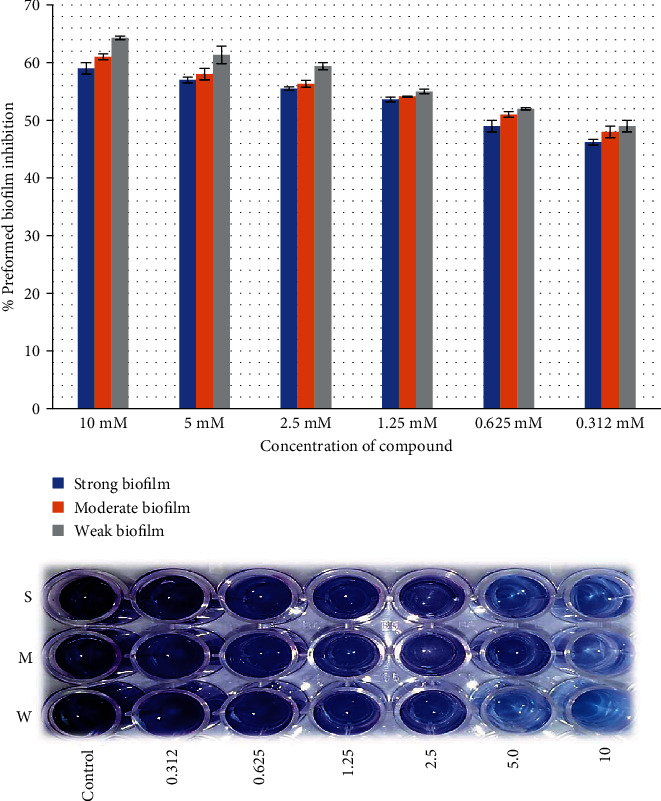
Effect of 2-methoxy-6-acetyl-7-methyljuglone on the biofilm formation of MDR *E. faecalis* as assessed by crystal violet staining. The bar graph indicates the percentage of biofilm attachment inhibition. S: strong; M: medium; W: weak biofilm producer.

**Figure 5:**
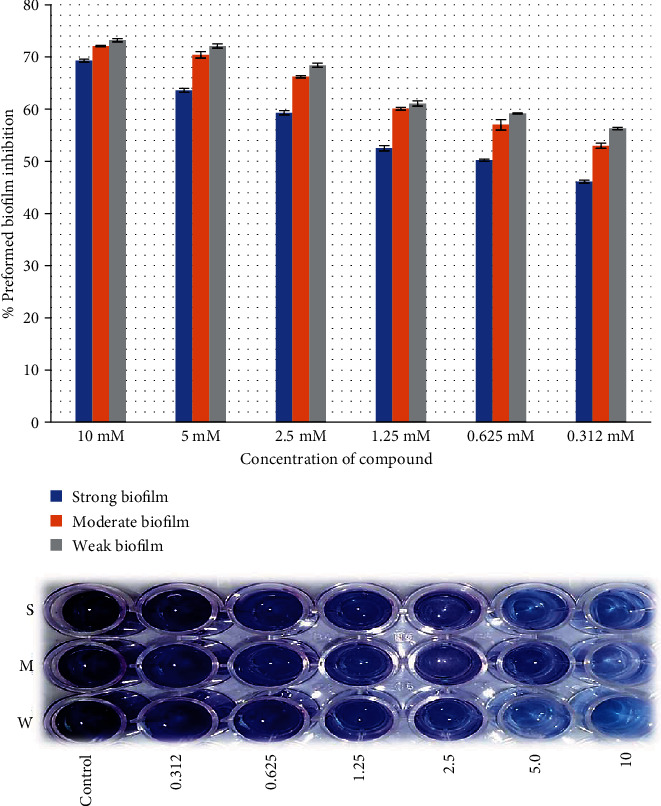
Effect of 2-ethoxy-6-acetyl-7-methyljuglone on the preformed biofilm of MDR *E. faecalis* as assessed by crystal violet staining. The bar graph indicates the percentage of preformed biofilm inhibition. S: strong; M: medium; W: weak biofilm producer.

Both juglones were applied against the preformed biofilm of *E. faecalis*; 2-ethoxy-6-acetyl-7-methyljuglone showed higher inhibitory efficacy than 2-methoxy-6-acetyl-7-methyljuglone with the same pattern. The highest activity was observed at highest concentration against low biofilm producers of the strain as compared to moderate and high biofilm producers (Figures 2–5).

### 3.7. Molecular Docking

**Figure 6:**
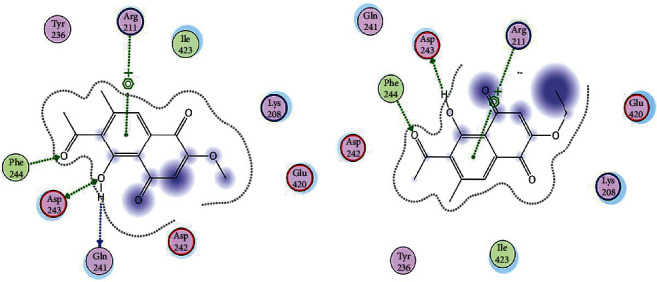
(a) 2D interaction of ligand molecule 2-methoxy-6-acetyl-7-methyljuglone with binding site residues of enterococcal surface protein (Esp); (b) 2D interaction of ligand molecule 2-ethoxy-6-acetyl-7-methyljuglone with binding site residues of enterococcal surface protein (Esp).

Both the ligand molecules (2-methoxy-6-acetyl-7-methyljuglone and 2-ethoxy-6-acetyl-7-methyljuglone) were docked into the binding site of the enterococcal surface protein. The results revealed that both 2-methoxy-6-acetyl-7-methyljuglone and 2-ethoxy-6-acetyl-7-methyljuglone were actively bound to the target site. In the case of 2-methoxy-6-acetyl-7-methyljuglone, a total of four hydrogen bond interactions were observed (Figure 6(a)). The binding site residues Arg211, Gln241, Asp243, and Phe244 were found to interact with ligand molecules, whereas ligand molecule 2-ethoxy-6-acetyl-7-methyljuglone was observed to interact with Arg211, Asp243, and Phe244, respectively (Figure 6(b)). The docking scores and binding energies of both ligand molecules are listed in Table 3.

A scoring function known as the generalized-Born volume integral/weighted surface area estimates the free energy required to bind ligand in a given pose. For each of the scoring functions, a lower score indicates a more advantageous pose. When conducting molecular docking research, the binding energies and the docking scores are the primary criteria used to determine which compounds are active and which are inactive.

**Figure 7:**
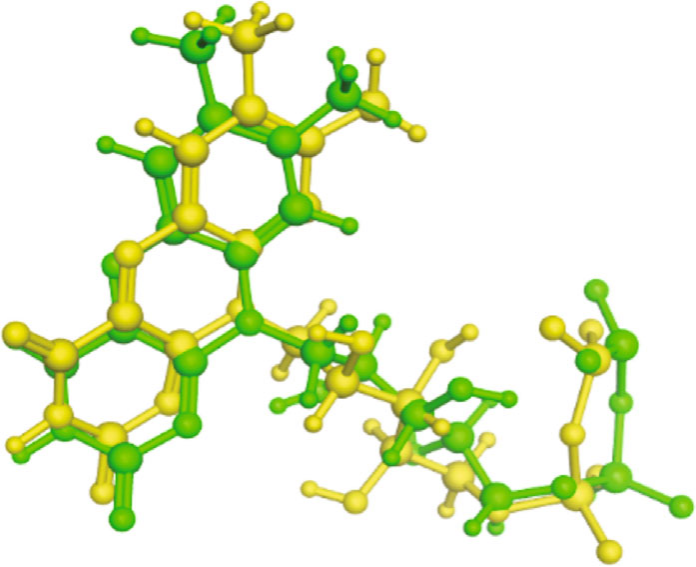
Yellow is the cocrystalized ligand and light green is the redocked pose of the same ligand.

For the validation of the MOE docking program, RMSD value was calculated. For this purpose, the cocrystallized ligand was removed from the protein and redocked in the same site. The found RMSD value was 1.3561 Å.  Figure 7 shows the validity of our docking method. It proves that MOE is reliable for docking studies.

## 4. Discussion

Enterococci are normal inhabitants of intestinal tract of humans and animals and constitute a predominant part of the environmental microbiota, found in food, water, sewage, soil, and plants. They are important opportunistic pathogens and pose a major threat for hospitalized and immunocompromised patients. Among enterococci, *Enterococcus faecalis* is the most prevalent and responsible for a number of infections, such as urinary tract infections, soft tissue and wound infections, bacteremia, peritonitis, and endocarditis [3].

On account of the high occurrence of nosocomial infections, *E. faecalis* is constantly exposed to multiple antibiotics, resulting in the emergence of its antibiotic resistance*. E. faecalis* possesses intrinsic resistance to some antibiotics including quinupristin-dalfopristin, lincosamides, cephalosporin, and low levels of aminoglycosides [18]. Over time, it has acquired resistance genes via HGT and mutation resulting in resistant phenotypes that are very difficult to treat. It has acquired resistance against quinolones, glycopeptides, and aminoglycosides, thereby leading to the emergence of MDR superbugs, which have resulted in few options for treatment [27].

In this study, the highest nonsusceptibility pattern was recorded against erythromycin (87%), ciprofloxacin (78%), and tetracycline (74%) by the Kirby-Bauer disk diffusion method. Moderate resistance was displayed against amoxicillin (61%) and gentamicin (39%). A similar study was performed by Shettigar et al., in which *E faecalis* showed high levels of resistance against erythromycin (94%), tetracycline (91%), and ciprofloxacin (89%) [28]. The highest level of susceptibility was recorded against linezolid (3%), vancomycin (13%), and fosfomycin (21%). A similar level of susceptibility was reported by Hussain et al. with vancomycin (98.13%) and linezolid (97.51%). The slight difference in susceptibility pattern of *E faecalis* was due to sample size, population, and the use of different antibiotics [18].

MDR strains of *E. faecalis* arise from the excessive use of antibiotics in livestock, plants, and humans. Continuous use of antibiotics eliminates the sensitive bacterial population, but the resistant ones remain alive, grow, and multiply [29]. MDR strains of *E. faecalis* increased substantially over the past decade and present a major healthcare burden today [30].

In our study, agar well diffusion assay was performed to evaluate antibacterial activity. A 15 ± 1 mm zone of inhibition was observed against the MDR and biofilm-producing strains and 16.2 ± 0.8 mm zone against ATCC 29212 by 2-methoxy-6-acetyl-7-methyljuglone. We found that 2-ethoxy-6-acetyl-7-methyljuglone produced a 17.3 ± 0.5 mm zone of inhibition against MDR strains and 18.1 ± 0.9 mm against ATCC 29212. Some studies have shown that juglones have the potential to target bacterial DNA, RNA cell wall, and cell membrane. A similar assay with very similar results was performed in India to check the antibacterial activity of a medicinal plant against *Escherichia coli*, *P. aeruginosa*, *Bacillus cereus*, and *staphylococcus aureuss* [22]. The clear zones of inhibition in our study supported such earlier work, wherein bioactive compounds extracted from medicinal plants were found to have strong antibacterial potential even against MDR strains.

The antibacterial activity of juglone derivatives was further evaluated through broth microdilution method. In current study, both molecules exhibited excellent antienterococcal activity against MDR *E. faecalis* and ATCC 29212 strain. Between the two compounds, 2-ethoxy-6-acetyl-7-methyljuglone exhibited stronger antienterococcal activity than 2-methoxy-6-acetyl-7-methyljuglone, with MIC values of 9.7 ± 3 *μ*M and 19.5 ± 2 *μ*M, respectively. In the current study, the MBC and MIC values were same for both the compounds which showed that these compounds were bactericidal at lower concentrations. A similar study was performed in Malaysia to determine the antibacterial activity of juglone derivatives against *E. coli*, *B. cereus*, *K. pneumoniae*, and S*S. aureuss* [30]. This indicated that juglone derivatives displayed a wide spectrum of antibacterial activity against different bacterial species.

In biofilms, bacterial populations grows in a community attached to biotic surfaces. It is an important phenomenon in the life cycle of microorganisms, which provides protection against harsh condition, desiccation, antiseptics, antibodies, antibiotics, and abrupt changes in pH. Biofilms are mostly composed of polysaccharides, hydrated exopolymeric substances, proteins, and nucleic acids [9, 12]. *E. faecalis* has strong biofilm formation potential that renders it 100−1000 times more resistant to antiseptics, antibodies, and antibiotics. In a majority of chronic infections caused by *E. faecalis*, biofilm-producing strains were involved. Biofilm formation not only provides protection but also enhances HGT giving rise to MDR strains and making the treatment more challenging [27, 31]. To control chronic infections and antibiotic resistance, it is thus crucial to focus on biofilm inhibition. While earlier studies confirmed the antibacterial activity of juglone derivatives, in this study, we tested their ability to inhibit the biofilm formation of *E. faecalis*.

The MDR strains of *E. faecalis* were sensitive to juglone derivatives used in the study in planktonic state. These compounds were also checked for their inhibition of biofilm attachment and preformed biofilms. The biofilm attachment inhibition rate of 2-ethoxy-6-acetyl-7-methyljuglone at 70% was found more promising as compared to that of 2-methoxy-6-acetyl-7-methyljuglone at 63.1% while their disruption rates of preformed biofilm were 69.3% and 59%, respectively. Wang et al. stated that juglones produced oxidative environment in the cell which resulted in cell wall disruption and increased cellular permeability leading to cell death. They also interfered with cell signaling leading to biofilm inhibition [32]. Our study found that the inhibitory effect of juglone derivatives reduced with a reduction in concentration. A similar study reported that 31.4% biofilm attachment inhibition and 7.5% of preformed biofilm inhibition were recorded against *E. faecalis* biofilm-producing strains [33]. Moreover, 2-ethoxy-6-acetyl-7-methyljuglone, which has an ethoxy group at the C-2 position, was found to have a slightly more potent activity than 2-methoxy-6-acetyl-7-methyljuglone, which has a methoxy group at the same position [13]. Qayyum et al. also evaluated the antibiofilm activity of quercetin against *E. faecalis* and recorded biofilm inhibition rates of 95%, 85%, and 70% at concentrations of 256, 128, and 64 mg/L, respectively [9]. Our study proves that juglone derivatives have a remarkable antibiofilm potential and can be used to eradicate biofilms, which represent a key cause of antibiotic resistance and chronic infections.

## 5. Conclusion

The findings of our study demonstrated that two juglone derivatives (2-methoxy-6-acetyl-7-methyljuglone and 2-ethoxy-6-acetyl-7-methyljuglone) isolated from *Reynoutria japonica* have very promising antibacterial activity against the MDR strains of *E. faecalis.* Furthermore, these compounds also have the potential to prevent biofilm formation by *E. faecalis*. According to our findings, 2-ethoxy-6-acetyl-7-methyljuglone has better antibacterial and antibiofilm activity as compared to 2-methoxy-6-acetyl-7-methyljuglone. Therefore, it is concluded that these natural compounds can be a good alternative to commercial antibiotics to treat infections caused by the MDR and biofilm-producing strains of *E. faecalis*.

## Figures and Tables

**Table 1:** Zones of inhibition (mm) for juglone derivatives against MDR and ATCC 29212 strains of *E. faecalis* at different concentrations.

Compounds	*Enterococcus faecalis* (MDR strain)				ATCC strain (29212)			
	10 mM	5 mM	2.5 mM	1.25 mM	10 mM	5 mM	2.5 mM	1.25 mM
2-Methoxy-6-acetyl-7-methyljuglone	15 ± 1	15 ± 0.5	14.3 ± 0.7	13.1 ± 0.9	16.2 ± 0.8	16 ± 0.7	15.3 ± 0.5	14.4 ± 0.6
2-Ethoxy-6-acetyl-7-methyljuglone	17.3 ± 0.5	17.1 ± 0.3	16.2 ± 0.5	15 ± 0.7	18.1 ± 0.9	18 ± 0.5	17.3 ± 0.4	16 ± 0.5
Gentamicin	15 ± 1 mm				17 ± 1 mm			
Dimethyl sulfoxide	−				−			

Data are expressed as the mean and SD of three independent experiments.

**Table 2:** Minimum inhibitory concentration of juglone derivatives against *E. faecalis* MDR biofilm positive strains and ATCC 29212 strain.

Compounds	MIC (*μ*M)	
	*Enterococcus faecalis* (MDR strain)	ATCC strain (29212)
2-Methoxy-6-acetyl-7-methyljuglone	19.5 ± 2	19.5 ± 1
2-Ethoxy-6-acetyl-7-methyljuglone	9.7 ± 3	9.7 ± 2

Data are expressed as the mean and SD of three independent experiments.

**Table 3:** Interacting residues, docking scores, and binding energies of ligand molecules.

S. no	Compound name	Interacting residues	Docking score	GBVI/WSA
1	2-Methoxy-6-acetyl-7-methyljuglone	Arg211, Gln241, Asp243, and Phe244	-12.4790	-25.684
2	2-Ethoxy-6-acetyl-7-methyljuglone	Arg211, Asp243, and Phe244	-11.7982	-24.547

## Data Availability

All data used to support the findings of this study are included within the article.
